# Intestinal Microbiome Changes in Fecal Microbiota Transplant (FMT) *vs.* FMT Enriched with *Lactobacillus* in the Treatment of Recurrent *Clostridioides difficile* Infection

**DOI:** 10.1155/2019/4549298

**Published:** 2019-12-28

**Authors:** Elvira Garza-González, Soraya Mendoza-Olazarán, Rayo Morfin-Otero, Andrea Ramírez-Fontes, Patricia Rodríguez-Zulueta, Samantha Flores-Treviño, Paola Bocanegra-Ibarias, Héctor Maldonado-Garza, Adrián Camacho-Ortiz

**Affiliations:** ^1^Universidad Autónoma de Nuevo León, Facultad de Medicina, Hospital Universitario “Dr. José Eleuterio González”, Monterrey, Nuevo León, Mexico; ^2^Universidad de Guadalajara, Hospital Civil de Guadalajara Fray Antonio Alcalde e Instituto de Patología Infecciosa y Experimental, Centro Universitario de Ciencias de la Salud, Guadalajara, Jalisco, Mexico; ^3^Infectious Diseases Service, Hospital General Manuel Gea González, Ciudad de Mexico, Mexico

## Abstract

**Aim:**

In this study, we conducted a comparative study to explore the differences in therapeutic efficacy and intestinal microbiome of fecal microbiota transplant (FMT) *vs.* FMT in addition with *Lactobacillus* (FMT-L) for treatment of recurrent *Clostridioides difficile* infection (R-CDI).

**Methods:**

We designed a double-blinded randomized comparative two-arm pilot multicenter study to assess the efficacy and impact in the intestinal microbiome of standard capsules of FMT *vs.* FMT-L enriched with 3 species of *Lactobacillus* for patients with R-CDI. A 90-day follow-up of 21 patients was performed, starting at the beginning of the study. From the selected patients, fecal samples were obtained at days 0, 3, 7, and 28 after treatment. Fecal samples and FMT were analyzed by 16S rRNA sequencing.

**Results:**

We included 21 patients (13 in the FMT group and 8 in the FMT-L group). Overall, both groups had a reduction in bowel movements per day, from 8.6 to 3.2 in the first 48 h (62.7% reduction, *p*=0.001). No severe adverse reactions or recurrences were recorded. Firmicutes were the most abundant phylum in donors. A low relative abundance of Proteobacteria was detected and mostly found in patients even at higher proportions than the donor. The donor's pool also had relatively few Bacteroidetes, and some patients showed a higher abundance of this phylum. Based on the ANOSIM *R* values, there is a significant difference between the microbial communities of basal samples and samples collected on day 7 (*p*=0.045) and at day 28 (0.041).

**Conclusion:**

Fecal microbiota transplant by capsules was clinically and genomically similar between traditional FMT and enriched FMT with *Lactobacillus* spp. Restoration of bacterial diversity and resolution of dysbiosis at days 7 and 28 were observed. Patients with a first episode of recurrence treated with FMT had an excellent response without severe adverse events; FMT should be considered as an early treatment during R-CDI.

## 1. Introduction

*Clostridioides* (*Clostridium*) *difficile* infection (CDI) is a complicated and recurrent communicable disease with limited proven treatment options. The spectrum of CDI is extensive, starting from asymptomatic carriage to severe diarrhea that may progress to pseudomembranous colitis and toxic megacolon [1, 2]. Recurrence occurs in up to 20–30% of patients [2], and subsequent treatments are less and less efficacious.

Fecal microbiota transplant (FMT) has been used in patients with recurrent events of CDI (R-CDI) with great success, and it is now a part of the IDSA guidelines for patients with multiple recurrences [3]. It has been used for the treatment of patients with R-CDI with an efficacy of 87–91% [4], and has shown to be a more effective treatment than vancomycin [5, 6].

*Lactobacillus* spp. have shown to serve as a preventive measure for the development of CDI in high-risk patients and in those who are recipients of antibiotic therapy. This effect is mainly attributable to their acidophilic effect and lowering of pH in the intestinal microenvironment [7, 8]. The addition of *Lactobacillus* spp. to FMT might enhance the efficacy of the latter by enhancing engraftment and therefore provides clinical advantages.

We conducted a comparative study to explore the differences in therapeutic efficacy and intestinal microbiome of FMT *vs.* FMT with the addition of *Lactobacillus* spp. for the treatment of R-CDI.

## 2. Materials and Methods

### 2.1. Informed Consent

The study was reviewed and approved by the Local Ethics Committee of the School of Medicine of the Universidad Autónoma de Nuevo León (approval IF-0016-3). All study participants provided informed written consent before study enrollment. The registry in Clinical Trials was performed right after the beginning of the protocol. All ongoing and related trials for this intervention are registered with the ClinicalTrials.gov Identifier NCT03804736.

### 2.2. Study Design and Settings

We designed a double-blinded randomized comparative two-arm pilot multicenter study to compare the efficacy and impact in the intestinal microbiome of standard FMT delivered by capsules (FMT) *vs*. FMT enriched with 3 species of *Lactobacillus* (FMT-L) for patients with recurrence of CDI.

We included patients from Hospital Universitario “Dr. José Eleuterio González” in Monterrey, Mexico, a 650-bed teaching hospital with an average of 25,000 annual discharges. We also included patients from Hospital Civil de Guadalajara Fray Antonio Alcalde, a 1000-bed tertiary-care teaching hospital with approximately 30,000 admissions each year and from Hospital Manuel Gea González, a 250-bed general hospital with 8750 admissions each year.

### 2.3. Definitions

Recurrence of CDI was defined as patients with a primary diagnosis ≥3 loose stools and positive test results for toxins using the ImmunoCard toxins A&B assay (Meridian Bioscience, Cincinnati, OH, USA) or real-time PCR (Cepheid Xpert C. difficile/Epi, Cepheid, Sunnyvale, CA) followed by an adequate response to treatment (absence of diarrhea, leukocytosis, and abdominal pain at the end of treatment) and developed new diarrhea (≤3 loose stools) within 8 weeks after treatment.

CDI resolution was considered by the absence of diarrhea, leukocytosis, and abdominal pain at the end of treatment. A new FMT was administrated if after 72 h from the first dose, the patient had an inadequate clinical response deemed as a reduction of less than 50% bowel movements and failure to improve consistency of stool.

### 2.4. Patient Selection

Participants were patients ≥16 years of age diagnosed with a recurrence of CDI. Participants had to be outpatients and had to have adequate oral intake. Pregnant patients, patients with GI derivative surgery, enteral fistulas, inflammatory bowel disease, or other causes of chronic diarrhea were excluded.

### 2.5. Randomization and Study Groups

In this pilot study, we proposed a sample size of 10 patients in each group. Once the patients had signed the informed written consent, they were randomized by the research coordinator in a 1 : 1 ratio to receive either FMT or FMT-L. Patients stopped any antibiotic at least 24 h before FMT.

Each patient received 15 frozen capsules PO every 12 h for a total of 4 doses (a total of 60 capsules). A patient identification (ID) was assigned to each participant, and samples collected were identified with this ID. The randomized patients received treatment according to the intervention they were allocated. Clinical data regarding bowel movements, fever, and abdominal pain as well as laboratory parameters were registered. Feces from each patient were collected before transplant on day 0 (sample A) and posttransplant on days 3 (sample B), 7 (sample C), and 28 (sample D).

Follow-up of patients was carried out from inclusion in the study up to 90 days. The study was ended when the minimum number of patients for each arm was completed.

### 2.6. Donor Selection and Preparation of Capsules

Healthy subjects over 18 years old, nonpregnant, with a body mass index of 20–25 kg/m^2^, a normal total blood count, and normal serum levels of liver enzymes were evaluated as described [9]. From the four selected donors, fresh stool collected was mixed with 15% (v/v) glycerol and stored at −70°C <1 h from recollection. At once, the feces were filtered three times through a sterile gauze to remove particles and filtrated, and they were then encapsulated in a double capsule (0 and 00 sizes) and again stored at −70°C until 10 min before ingestion. Capsules for the FMT-L were additionally enriched with a mixture containing *Lactobacillus casei, Lactobacillus acidophilus*, and *Lactobacillus rhamnosus* with a total of 10^8^ CFU from each species per capsule. *Lactobacillus* spp. used in the study were provided by our own collection of typed isolates.

### 2.7. Microbiome Analysis

The semiconserved V4 region of the16S rRNA gene was amplified using described primers [10]. The PCR amplicons were sequenced on a MiSeq instrument (Illumina) at Molecular Research LP (Shallowater, Texas, USA) following manufacturer's protocols. The Q25 sequence data derived from the sequencing process were processed using a proprietary analysis pipeline (http://www.mrdnalab.com, MR DNA, Shallowater, TX). Sequences were depleted of barcodes and primers, and then short sequences <200 bp, sequences with ambiguous base calls, and sequences with homopolymer runs exceeding 6 bp were removed. Sequences were then denoised, and chimeras were removed.

Operational taxonomic units (OTUs) were defined after removal of singleton sequences, clustering at 3% divergence (97% similarity). OTUs were then taxonomically classified using BLASTn against a curated NCBI database and compiled into each taxonomic level into both “counts” and “percentage” files.

### 2.8. Statistical Analysis

A descriptive analysis with 95% confidence intervals was performed. For dichotomous variables, a *χ*2 test or Fisher's exact test was used. For continuous variables, we used the Wilcoxon sum rank test. A value of *p* < 0.05 was considered statistically significant. We used SPSS software ver. 20.0 for analysis.

For microbiome analysis, statistical analysis was performed using a variety of computer packages including XLstat, NCSS 2007, “*R*,” and NCSS 2010. Alpha and beta diversity analysis were conducted as described previously using Qiime 2 (https://peerj.com/preprints/27295/). Significance reported for any analysis is defined as *p* < 0.05.

## 3. Results

### 3.1. Population and Clinical Response

A total of 30 patients were screened, and of those, 21 fulfilled the enrollment criteria. The average age was 61 years (range 17–91), and 57.1% were female. Baseline bowel movements were 8.6 (range 3–20) per day, and the initial Bristol score was 6.5 (range 5–7). Patients were randomized to receive FMT or FMT-L (Table 1).

Overall, both groups of patients had a reduction in bowel movements per day from 8.6 to 3.2 (62.7% reduction, *p*=0.001) in the first 48 h and the Bristol score was also reduced from 6.5 to 5.4 in the same period, but did not reach statistical significance (*p*=0.15). Patients with a first R-CDI accounted for 76.2% of the totality of enrolled patients.

There were no differences in baseline characteristics between the groups and in the clinical response rate.

Adverse events were recorded in 42.3% of patients and were predominantly burping (14.8%), constipation (19%), and vomiting (9%). No severe adverse events were recorded. One patient died on the eleventh day of follow-up due to an acute myocardial infarction not related to the study intervention, and that patient had an excellent clinical response during the short follow-up with no adverse events after the intervention.

Patients were clinically followed for an average of 90 days, and no recurrences were reported. Furthermore, they were followed up by a telephone call after 24 weeks and none of them reported symptoms consistent with the development of recurrence.

### 3.2. Microbiome Analysis

Samples from 13 patients (8 FMT and 5 from FMT-L) were subjected to metagenomic analysis. After stringent quality sequence curation, a total of 3,494,550 sequences were parsed and 3,222,306 were then clustered. Finally, 3,219,912 sequences were identified within the bacteria, and the *Archaea* domains were utilized for the final microbiome analysis. The average reads per sample was 89,442. For alpha and beta diversity analysis, samples were rarefied to 20,000 sequences. Data were evaluated in a multivariate manner to determine the changes between groups.

**Figure 1:**
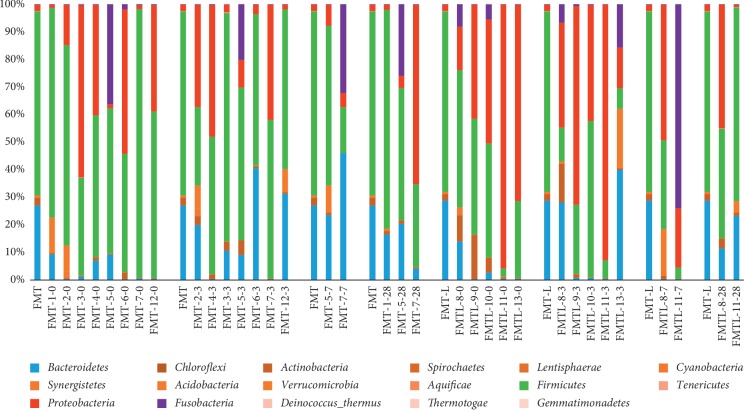
Average bacterial composition at the phylum level. Distribution of bacterial families in the fecal microbiome transplant (FMT) group and the *Lactobacillus*-FMT (L-FMT) group. The label of each sample (e.g., FMT-5-0) denotes treatment (FMT or L-FMT), the assigned number of each patient, and days on treatment (0 = baseline, 3 = 3 days on treatment, 7 = 7 days on treatment, and 28 = 28 days of treatment). To facilitate comparison and visualization, the distribution of bacterial families of the donors is presented for the FMT group and the L-FMT group.

Average bacterial composition at the phylum level is shown in Figure 1. Few changes were detected, and Firmicutes, Bacteroidetes, and Proteobacteria were the most abundant phyla. Interestingly, Firmicutes were the most abundant phylum in donors; and the proportion was higher in some patients at days 3 and 7 especially in the FMT group.

**Figure 2:**
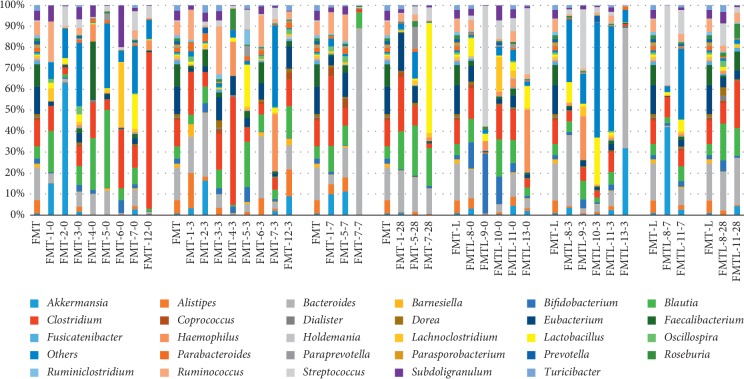
Average bacterial composition at the genera level. Distribution of bacterial genera in the fecal microbiota transplant (FMT) group and the *Lactobacillus*-FMT (L-FMT) group. The label of each sample (e.g., FMT-5-0) denotes treatment (FMT or L-FMT), the assigned number of each patient, and days on treatment (0 = baseline, 3 = 3 days on treatment, 7 = 7 days on treatment, and 28 = 28 days of treatment). To facilitate comparison and visualization, the distribution of bacterial families of the donors is presented for the FMT group and the L-FMT group.

Furthermore, the donors' sample had a low relative abundance of Proteobacteria, and this phylum was detected in most patients even at higher proportions than the donor. The donor's pool also had relatively few Bacteroidetes, and some patients showed higher abundance for this phylum. Regarding the *Lactobacillus* genus, the behavior was unexpected although the difference in diversity was higher in L-FMT compared to FMT because of enrichment; after transplant there were a greater number of OTUs in the FMT (2224 range 47–13776) group vs. the L-FMT (521 range 62–1987) group although it failed to reach statistical significance (Figure 2) (*p*=0.52).

### 3.3. Alpha Diversity of Samples

**Figure 3:**
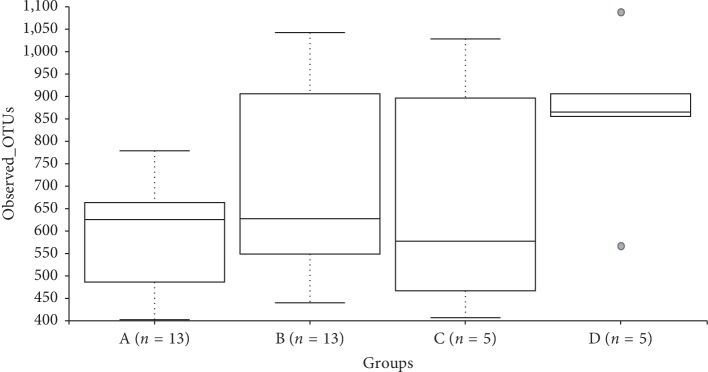
Observed OTUs boxplot and Kruskal–Wallis pairwise comparisons.

**Figure 4:**
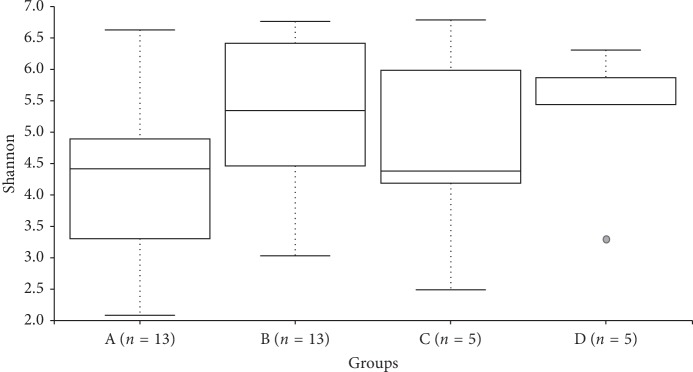
Shannon diversity boxplot.

Statistical comparisons of observed OTUs and Shannon Diversity indices for each sample group were conducted using Kruskal–Wallis pairwise comparisons (Figures 2 and 3). The comparisons between groups in each of these alpha diversity metrics only detected one significant difference. The number of observed OTUs is significantly higher in Group D compared to the number of OTUs detected in Group A (*p*=0.016). Comparisons between the Shannon diversity indices of Groups A and D yielded no significant difference (Figure 4) (*p*=0.085).

### 3.4. Beta Diversity of Samples

**Figure 5:**
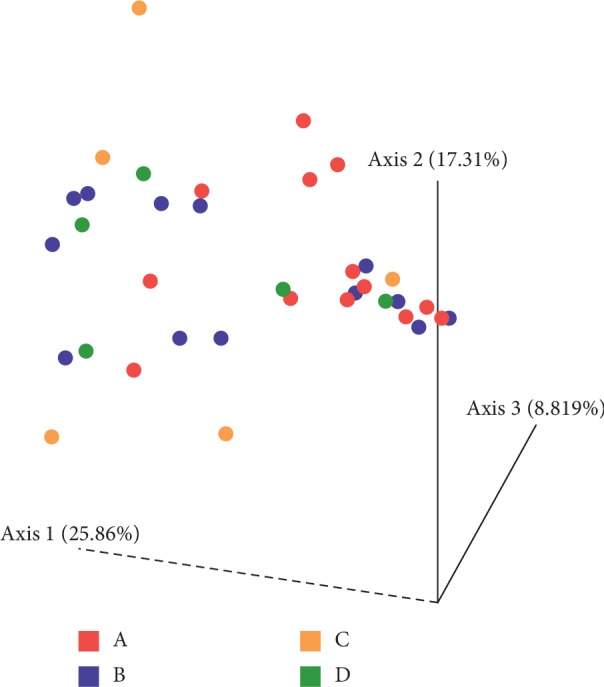
Principal coordinate plot of weighted UniFrac data. Colors keyed on the group: A (red), B (blue), C (orange), and D (green). Primary vector explains 25.8% of the variation between the groups. The first 3 vectors together exhibit 51.9% of the variation among the groups.

The microbial community structure was analyzed using weighted UniFrac distance matrices. Principal coordinate analysis plots were used to visualize the data in these matrices, and pairwise analysis of similarities (ANOSIM) was utilized to determine if there were any significant differences between the microbial communities (Figure 4). There appears to be no phylogenetic assemblage amongst any sample group that is significantly different from the remaining groups. However, based on the ANOSIM *R* values there is a significant difference between the microbial communities of Groups A and C (baseline and day 7) (0.045) as well as Groups A and D (baseline and day 28) (Figure 5) (0.041).

## 4. Discussion

In the current study, we explored the clinical and genomic differences in patients with R-CDI treated with 2 modalities of FMT, traditional and enriched with *Lactobacillus*.

The delivery of FMT by oral capsules hinders some diversity in the proportion of engraftment. Allegretti et al. studied the mode of delivery of FMT, finding no difference in the total clinical efficacy although greater engraftment was seen when the delivery was in the colon opposed to the upper gastrointestinal tract [11]. Our methods composed of a double capsule is similar to the used by the authors for the colon delivery and had a higher cure rate although their population had an average of 3 or more recurrences as opposed to ours that had predominantly first and second recurrences.

Current treatment guidelines recommend FMT as a therapeutic modality only after the second or third recurrence of CDI [3]. We, however, included patients with a first recurrence (over 75% of our patients) and patients on their second recurrence (almost 15%) with less than 10% on their third recurrence. Patients on their first and second recurrence had an excellent clinical response rate, and only one patient on the FMT group that was suffering from a first recurrence did not meet the study criteria for resolution at 72 h after FMT; the same patient had a retransplant, and clinical resolution was achieved without recurrence at six months. These results challenge the fact that FMT should be considered after the second recurrence since there was an overwhelming response rate in patients with the first recurring episode without severe adverse effects. This might be related to lower dysbiosis during the first and second recurrences.

The behavior of the *Lactobacillus* genus was unexpected although there was no statistical difference between the groups that *per se* is bewildering: We expected a discrepancy between both groups with regards to *Lactobacillus* spp. quantities favoring the FMT-L group; we speculate that 3 factors may have played a role in the discrepancies, and the first is exposure of the gut microbiome to community *Lactobacillus* spp. via food intake (although we encourage patients not to take probiotics without disclosure, we did not control this factor) and that the patients already had an abundant proportion at baseline. The second factor was that we did not analyze the totality of the samples from both groups and this possibly had selection bias, and finally that the amount of *Lactobacillus* spp. that was used for enrichment in relation to the quantity of the whole microbiota was too small to make a significant difference.

At the phylum level, some variation was noted, and typical engraftment patterns were not seen in all patients; Firmicutes were an abundant phylum in donors with a relatively low proportion of Proteobacteria and Bacteroidetes which in some patients were abundant after FMT. Studies have shown that engraftment and similarity to the donor's microbiome does not always match; furthermore, there is significant variation when the FMT is autologous or heterologous [12]. These variations of engraftment to our knowledge are the first description using a heterologous pool of feces from various donors.

It has been reported that administration of FMT to R-CDI patients results in restoration of bacterial diversity and resolution of dysbiosis, and that shift in diversity is incremental rather than immediate (Gut Microbes. 2017; 8 (3): 276–288). In our study, based on the ANOSIM values, a significant difference between the microbial communities of baseline and day 7 groups was detected (*p*=0.045) as well as between baseline and 28 days group (*p*=0.041).

Some limitations of our study include a relatively small sample size and that we did not analyze all the patient's samples for microbiome composition. Clinical response was extremely similar, and no greater difference in the composition was registered; thus, we estimate that with a bigger sample size the characteristics will be similar.

## 5. Conclusion

Fecal microbiota transplant by capsules was clinically and genomically similar between traditional FMT and enriched FMT with *Lactobacillus* sp, with significant changes in the restoration of bacterial diversity and resolution of dysbiosis at days 7 and 28 in the whole patient analysis.

Patients during the first episode of recurrence treated with FMT had an excellent response without severe adverse events; FMT should be considered as an earlier treatment during R-CDI.

## Figures and Tables

**Table 1:** Demographics, clinical, and treatment characteristics of both groups.

Characteristics		Combined (*n* = 21)	FMT (*n* = 13)	FMT-L (*n* = 8)	Univariate *p* value
Demographics					
Age mean (range)		58.9 (17–91)	56.8 (17–91)	62.4 (53–77)	0.764
Female gender *n* (%)		12 (57.1)	8 (61.5)	4 (50)	0.673

Clinical data					
Current recurrence episode, *n* (%)	First	16 (76.2)	12 (92.3)	4 (50)	**0.045**
	Second	3 (14.2)	0 (0.0)	3 (37.5)	0.133
	Third	2 (9.5)	1 (7.7)	1 (12.5)	0.421

Previous antibiotic use		21 (100)	13 (100)	8 (100)	NA
Third-generation cephalosporin		11 (52.3)	6 (46.1)	5 (62.5)	0.659
Fluoroquinolone		7 (33.3)	5 (38.4)	3 (37.5)	0.841
Clindamycin		5 (23.8)	4 (30.7)	2 (25.0)	0.776
Other		5 (23.8)	5 (38.4)	4 (50.0)	0.94

Bowel movements per day, mean (range)	Basal	8.6 (3–20)	8.9 (3–20)	8.4 (3–12)	0.779
	Day 2	3.2 (0–9)	3.3 (0–9)	3.25 (2–5)	0.947
	Day 90	1.4 (0–3)	1.4 (0–3)	1.5 (0–3)	0.843

Bristol score, mean (range)	Basal	6.5 (5–7)	6.46 (5–7)	6.37 (5–7)	0.803
	Day 2	5.4 (1–7)	5.2 (1–7)	5.87 (4–7)	0.342
	Day 90	3.6 (0–5)	3.0 (1–4)	4.75 (4–5)	0.073

Total body weight, mean (range)	Basal	63 (38–94)	60.7 (38–94)	71.5 (49–89)	0.216
	Day 90	66 (39–94)	63.3 (39–94)	74.3 (50–93)	0.241

Recurrence after FMT	1 (4.8%)	1 (7.7)	0 (0.0)	0.421	
Minor adverse events, *n* (%)	Burping	3 (14.2)	2 (15.4)	1 (12.5)	0.854
	Constipation	4 (19)	4 (30)	0 (0.0)	0.241
	Vomiting	2 (9)	1 (7.7)	1 (12.5)	0.075

Severe adverse events, *n* (%)		0 (0)	0 (0.0)	0 (0.0)	NA

The bold value indicates that it is statistically significant because it is lower than 0.05.

## Data Availability

The data used to support the findings of this study are available from the corresponding author upon request.
